# Mining the Mind: Linear Discriminant Analysis of MEG Source Reconstruction Time Series Supports Dynamic Changes in Deep Brain Regions During Meditation Sessions

**DOI:** 10.1007/s10548-021-00874-w

**Published:** 2021-10-15

**Authors:** Daniela Calvetti, Brian Johnson, Annalisa Pascarella, Francesca Pitolli, Erkki Somersalo, Barbara Vantaggi

**Affiliations:** 1 Department of Mathematics, Applied Mathematics and Statistics, Case Western Reserve University, 10900 Euclid Avenue, Cleveland, OH 44106 USA; 2 Istituto per le Applicazioni del Calcolo “Mauro Picone” - CNR, Via dei Taurini 19, 00185 Rome, Italy; 3 Department of Basic and Applied Sciences for Engineering, University of Rome “La Sapienza”, Via Scarpa 16, 00161 Rome, Italy; 4 Department MEMOTEF, University of Rome “La Sapienza”, Via del Castro Laurenziano 9, 00161 Rome, Italy

**Keywords:** MEG inverse problem, Activity map, Meditation, Spectral analysis, Linear discriminant analysis, Deep sources

## Abstract

Meditation practices have been claimed to have a positive effect on the regulation of mood and emotions for quite some time by practitioners, and in recent times there has been a sustained effort to provide a more precise description of the influence of meditation on the human brain. Longitudinal studies have reported morphological changes in cortical thickness and volume in selected brain regions due to meditation practice, which is interpreted as an evidence its effectiveness beyond the subjective self reporting. Using magnetoencephalography (MEG) or electroencephalography to quantify the changes in brain activity during meditation practice represents a challenge, as no clear hypothesis about the spatial or temporal pattern of such changes is available to date. In this article we consider MEG data collected during meditation sessions of experienced Buddhist monks practicing focused attention (Samatha) and open monitoring (Vipassana) meditation, contrasted by resting state with eyes closed. The MEG data are first mapped to time series of brain activity averaged over brain regions corresponding to a standard Destrieux brain atlas. Next, by bootstrapping and spectral analysis, the data are mapped to matrices representing random samples of power spectral densities in $\alpha$, $\beta$, $\gamma$, and $\theta$ frequency bands. We use linear discriminant analysis to demonstrate that the samples corresponding to different meditative or resting states contain enough fingerprints of the brain state to allow a separation between different states, and we identify the brain regions that appear to contribute to the separation. Our findings suggest that the cingulate cortex, insular cortex and some of the internal structures, most notably the accumbens, the caudate and the putamen nuclei, the thalamus and the amygdalae stand out as separating regions, which seems to correlate well with earlier findings based on longitudinal studies.

## Introduction

Meditation practices are widely recognized as potentially powerful means for promoting physical and mental health through their acclaimed capability to reduce stress and anxiety (Hofmann et al. 2011), increase concentration and cognitive performance (Lutz et al. 2009), and improve self-image (Koole et al. 2009). The benefits of meditation claimed by practitioners include, but are not limited to, the regulation of mood and emotions, and the improvement of the concentration power (Basso et al. 2019; Zhang et al. 2019). Since the effects of the meditation practice are often based on self-reporting, questions have been raised about the bias towards positive effects (Lutz et al. 2009; Fox et al. 2014), making it hard to quantify and assess the clinical potential of meditation. In recent years, numerous studies were conducted to investigate the effects of meditation on the brain (Newberg 2014), however, understanding its influence is still far from complete. The key elements across meditation practices are attention control, emotion regulation and self-awareness (Tang et al. 2015; Grecucci et al. 2015). This motivates researchers to look for long-term or short-term changes in the associated brain regions. To determine whether the meditation benefits are a placebo perception or the result of structural and functional changes in the brain, quantitative evidence to support the claims is needed, an approach advocated also by the Dalai Lama.

In recent years, stress reducing lifestyle changes have been strongly advocated as an alternative to pharmacological intervention. As part of this movement, mindfulness meditation intervention has been considered as a potential tool, and programs such as mindfulness-based stress reduction (MBSR) (Kabat-Zinn 1990, 2003; Baer 2003) and mindfulness-based cognitive therapy (MBCT) (Segal et al. 2018) have emerged to deal with a gamut of mental disorders and behavioral problems. Concurrently, the number of scientific publications investigating whether, and if so, how, meditation changes the brain, have increased at a steady rate.

There is a body of literature on anatomical and morphological changes in the brain that meditation practice elicits. Cross-sectional studies look for differences between the brains of meditators and those of control groups, in particular, morphological and physiological differences in the brain structure, including cerebral blood flow (Newberg et al. 2001), microstructure of the white matter (Laneri et al. 2016), cortical thickness and gray matter density (Lazar et al. 2005), volume (Luders et al. 2013; Luders and Kurth 2019) and gyrification (Luders et al. 2012) in the brain regions that are believed to play a central role in meditation. In longitudinal studies, long term temporal changes due to the meditation practice are monitored (Gotink et al. 2016). For a recent meta-analysis, we refer to (Pernet et al. 2021). In long term studies, confounding factors such as changes in lifestyle accompanying the decision to engage in extensive meditation practice cannot be neglected, in particular because the test groups often involve professional meditators, who have dedicated years to this lifestyle changing practice. To distinguish between meditation related changes and possible confounding causes, functional brain imaging modalities provide a potential avenue towards a better understanding of the effects of meditation.

Functional brain imaging modalities such as functional magnetic resonance imaging (fMRI), electroencephalography (EEG) and magnetoencephalography (MEG) provide a potential way to track the changes in brain activity patterns elicited by the meditation. This study aims at identifying brain regions where the neural activity changes as the subject engages in meditative practices. The subjects considered in this study are professional meditators from the Theravada Buddhist tradition, and the data are collected during sessions corresponding to three brain states: The reference state with eyes closed, focused attention meditation (Samatha), and open monitoring meditation (Vipassana). In order to pinpoint the brain regions susceptible to activity changes when entering or exiting the meditative state, we first solve the MEG inverse problem, interpreting the biomagnetic signals in terms of the impressed currents in the brain, and we then compute the aggregate activity over individual brain regions defined by a chosen brain atlas. The MEG inverse problem is solved using a hierarchical Bayesian algorithm particularly suitable for analyzing brain activity in deep brain regions (Calvetti et al. 2015, 2019).

The brain atlas used in this study combines the cortical Destrieux atlas (Destrieux et al. 2010) with an atlas of subcortical structures (Attal et al. 2012). We use the estimated aggregate activity in the brain regions to generate large samples of the realizations of spectral densities over the $\theta$, $\alpha$, $\beta$, and $\gamma$ spectral bands and carry out a spectral analysis. Applying this procedure to each brain state, we generate three large annotated data sets for each brain state and individual. We test the separability of the data sets with the linear discriminant analysis (LDA). The LDA is a classical dimension reduction method that seeks to find the directions in the data space that best separate the annotated data sets. As the attributes of the data correspond to the different brain areas included in the atlas, the separating directions can be interpreted in terms of brain regions, and the most significant components carry information about those that are more relevant for the separation of the states. Our analysis shows consistency between different meditation sessions of single individuals, and to some extent also between different individuals indicating that the method is able to identify the key brain regions involved in the meditative process. The findings are also in line with a number of meditation studies reported in the literature, as pointed out in the discussion section.

### Our Contributions

The main methodological novel contribution of this article is the combination of the MEG inverse solver and the data analysis methods. Rather than applying the LDA directly to magnetometer data, or the spectral data, the MEG inverse problem is first solved time slice by time slice, and the resulting brain activity time series are aggregated over the regions of interest. A novel way to generate spectral band resolved randomized data sets through bootstrapping, windowing, and Fourier analysis is introduced, and LDA is then applied to the preprocessed data. The significant advantage of this processing is that, unlike any previously proposed use of LDA, our approach is highly interpretable, allowing us to identify brain regions that contribute to the separation between different states. The extensive literature review included in the article underlines the consistency of our findings with previous findings based on anatomical analysis of the effects of meditation.

## Materials and Methods

The data consist of MEG recordings of two professional meditators in the Theravada Buddhist tradition with an average of over 15,750 h of meditation practice, recruited from the Santacittarama monastery in Italy. In addition to the regular daily 2 h meditation practices, the monks take part in regular intensive meditation retreats, practicing two meditation styles: focused attention (Samatha) and open monitoring (Vipassana) meditation. In addition to the data collected during the two meditation styles, reference data with no active meditation with eyes closed (rest) are provided. Preceded and followed by a period of 3 min of resting eyes closed, the meditators performed a block of 6 min of Samatha meditation, followed by 6 min of Vipassana, while sitting in the MEG scanner eyes closed. The cycle was repeated several times, the switch of protocol being instructed to the meditators through an auditory word signal consisting of the name of the protocol. A more detailed description of the data acquisition process, the selection of subjects, and the MEG device can be found in Marzetti et al. (2014).

Theravada (’the school of elders’) represents the oldest of the Buddhist traditions, and according to many Buddhist scholars (see, e.g., Bodhi 2005) the most original and closest to the original Buddhist teachings and the very foundation of Buddhist practice. The meditation practice represents a form of what currently is referred to as mindfulness meditation, the term mindfulness, indicating ’constant presence of mind’, a translation of the word ’sati’ in the Theravada Pali canon and originally proposed by T.H. Rhys Davids in late nineteenth century (Gethin 2011). The two main types of Buddhist meditation are Samatha meditation, to develop serenity and calm, and Vipassana meditation, to develop insight. All forms of Samatha start with an object on which the meditator concentrates on, most commonly the breath, which is believed to be most closely linked to the mind (Bodhi 2000). This meditation is referred to as Anapanasati, or ’mindfulness of breathing’ (Nananmoli 1998). While several different religious traditions contain some form of serenity practice, e.g., in the form of prayer, the most distinctive feature of the Buddhist practice is the Vipassana meditation, or insight meditation. The insight meditation is objectless meditation, and its non-judgmental observation of the mind processes has the goal of gaining a direct insight into the ultimate nature of reality, including the mind itself. This insight is closely related to the core Buddhist philosophy of learning to discern the unsatisfactory and non-substantial (’dukkha’) (Piyadassi 1991). The secular versions of mindfulness meditation (see, e.g., Kabat-Zinn 1990) aiming at stress reduction and pain management were largely derived from the Buddhist meditative practices (Nyanaponika 1962). In the secular context, these two fundamentally different meditation styles, while falling under the generic umbrella of mindfulness meditation, are often distinguished as focused attention (FA) and open-monitoring (OM) meditation (Raffone and Srinivasan 2010). As discussed in Lutz et al. (2015), there is no consensus about the precise definition of mindfulness, however, in the current context of psychology and cognitive neuroscience, the Buddhist term mindfulness corresponds to the psychological process of meta-awareness, defined in Dahl et al. (2015) as heightened awareness of the processes of consciousness, including the process of thinking, feeling, and perceiving. In a larger landscape of meditational practices, the meditation styles considered here fall under the family of deconstructive practices, discussed in detail in the cited articles and references therein.

### From MEG Data to Activity Vectors

The raw MEG data were pre-processed using independent component analysis in order to remove cardiac and eye movement artifacts. This preprocessing step was performed within the open source package NeuroPycon Meunier et al. (2020) based on MNE Python routine (Gramfort et al. 2013). The MRI data of the subjects were segmented with Freesurfer (Dale et al. 1999) and imported in Brainstorm (Tadel et al. 2011) to generate a source model including both cortical surface and subcortical regions. Subsequently, the time series data were processed by the iterative alternating sequential (IAS) hierarchical Bayesian algorithm, which is described in detail in Calvetti et al. (2015), and analyzed further in Calvetti et al. (2019), resulting into a time resolved dipole field time series $\{Q(t) = [\mathbf {q}_1(t), \ldots , \mathbf {q}_M(t)]\}$ over the source space of cardinality $M=20,000$. To reduce the dimensionality of the brain signals, we generate an activity map over a selected brain atlas by calculating the aggregate amplitude of the dipoles included in each brain region (BR) in the atlas. The choice of using aggregate instead of average amplitude over each brain region is motivated by the belief that the activity patches are more or less of invariant size, and averaging would bias the activity towards small brain regions. We define the *brain region activity level indicator* (BR-ALI) vector,

1 $$\begin{aligned} \mathbf{a}(t) = \left[ \begin{array}{c} a_1(t) \\ \vdots \\ a_n(t)\end{array}\right] , \quad a_\ell (t) = \text{sum}\{\Vert \mathbf {q}_j(t)\Vert \mid v_j\in {{\mathcal {R}}}_\ell \}, \end{aligned}$$

where $\Vert \mathbf {q}_j\Vert$ denotes the intensity of the resolved dipole source at the source space vertex $v_j$, and ${{\mathcal {R}}}_\ell$ is the $\ell$th parcel of source space vertices corresponding to the $\ell$th BR in the atlas. In this work, we subdivide the cortical region into 74 BRs per hemisphere according to the Destrieux atlas (Destrieux et al. 2010). Following (Attal and Schwartz 2013), we parcellate the internal structures into 8 BRs for each hemisphere, plus the brainstem. The total number of BRs in our analysis is $n = 165$. Observe that because no subsampling of the time series is performed, at the end of this process we have a time series of the BR-ALI vectors sampled with approximately one millisecond time resolution. The data acquisition provides several vectors of independently processed sequences of three different protocols (rest, Samatha, Vipassana), originating from different individuals. In this paper, the analysis is limited to two meditators, referred to as Subject 1 and Subject 2, for which the inverse reconstruction was available.

### Bootstrapping, Periodogram Samples, and Power Spectra

The time dependent BR-ALI vectors $\mathbf{a}(t)$ corresponding to a selected protocol and subject are given at discrete times $t = t_k = k\varDelta t$. The sampling frequency is denoted by $\nu _s = 1/\varDelta t$. With $\varDelta t \approx 1\, \text{ms}$, the sampling frequency is $\nu _s \approx 1000\, \text{Hz}$. In our calculations, we use the cosine transform, however, to conform with standard definitions, the complex exponential notation is used below.

To analyze the BR-ALI vectors in the frequency domain, we start by recalling the definition of the power spectral density (PSD) of a scalar random signal $Y = (Y(0),Y(1),\dots ,Y(N-1))$ of length *N*,

2 $$\begin{aligned} \text{PSD}(Y)(\nu ) = {{\mathbb {E}}}\left\{ \frac{1}{N} \left| \sum _{j=0}^{N-1} Y(j) e^{-i2\pi \nu j\varDelta t}\right| ^2\right\} \approx \frac{1}{p} \sum _{\ell =1}^p \varPhi ^{\ell }(\nu ), \end{aligned}$$

where the expectation (${{\mathbb {E}}}$) is computed over periodograms of *p* realizations of the signal (Stoica and Moses 2005), denoted by $y^{(\ell )} =(y^{(\ell )}(0),y^{(\ell )}(1),\ldots ,y^{(\ell )}(N-1))$, $1\le \ell \le p$, and

3 $$\begin{aligned} \varPhi ^{(\ell )} (\nu ) = \frac{1}{N} \left| \sum _{j=0}^{N-1} y^{(\ell )}(j) e^{-i2\pi \nu j\varDelta t}\right| ^2, \quad 1\le \ell \le p. \end{aligned}$$

**Fig. 1:**
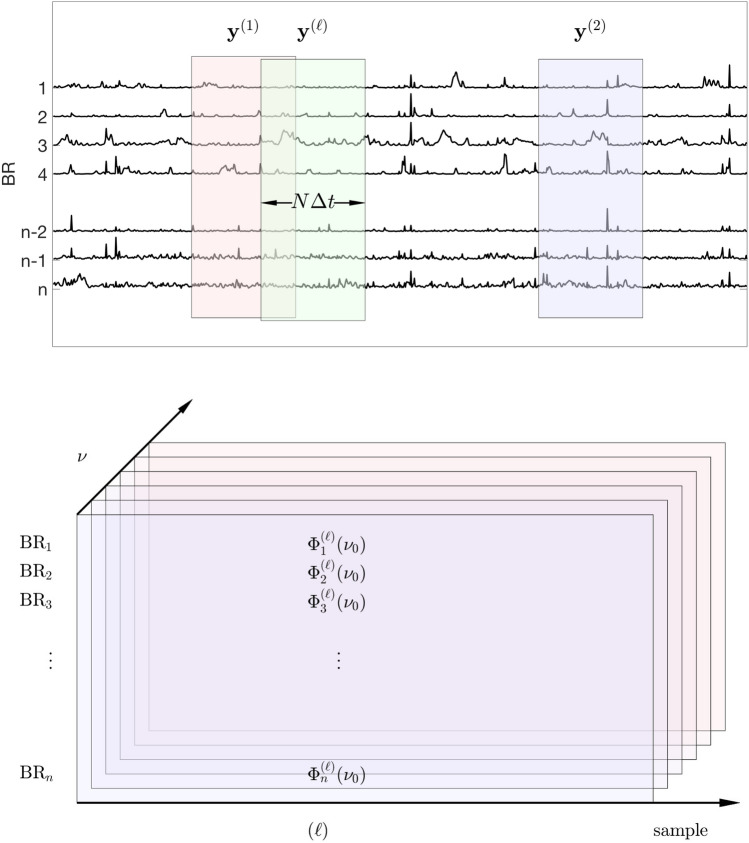
Top: A schematic picture of the bootstrap process. The matrices $\mathbf{y}^{(\ell )}$ with entries $y^{(\ell )}_m(k)$ as defined in Eq. (4) comprise random cuts of fixed width $N\varDelta t$ from the brain activity time series matrix. The sample vectors are Fourier transformed row-by-row, and the periodogram matrices are row-wise scaled squared amplitudes of the transforms, each row corresponding to a BR, the columns referring to the frequencies. Bottom: Organization of the bootstrapped periodogram data into a three-dimensional array. Each array corresponds to one meditation protocol and one meditator. The power spectra are computed by averaging over the samples (dimension 2), and the spectral density sample matrices are computed by integrating (or in practice, summing) over a given spectral band (dimension 3)

For our analysis, starting from the BR-ALI vectors with *n* components corresponding to *n* BRs, we define a sample of periodogram vectors using bootstrapping: Given a sampling window of length $T_s = N\varDelta t$, where *N* is an integer, we denote by

4 $$\begin{aligned} \mathbf{y}^{(\ell )}(k)= & {} \left[ \begin{array}{c} y^{(\ell )}_1(k) \\ \vdots \\ y^{(\ell )}_n(k)\end{array}\right] \nonumber \\= & {} \mathbf{a}(t^{(\ell )} + k \varDelta t)\in {{\mathbb {R}}}^n, \quad k=0,1,\ldots ,N-1, \end{aligned}$$

a random sample of length $T_s$ of the time series, where the initial value $t^{(\ell )}$, drawn randomly from the interval of observation, coincides with one of the discretization point. Figure 1 elucidates the idea of the bootstrapping process.

In the computations, we restrict the frequencies $\nu$ to a discrete grid,

5 $$\begin{aligned} \nu = \nu _k = k/T_s, \quad 0\le k\le N-1, \end{aligned}$$

and, for each fixed frequency $\nu _k$, define the bootstrapped periodogram matrices of size $n\times p$,

6 $$\begin{aligned} \varPhi _m^{(\ell )}(\nu _k)= & {} \frac{1}{N} \left| \sum _{j=1}^N y^{(\ell )}_m(j) e^{-i2\pi \nu _k j\varDelta t}\right| ^2 \nonumber \\= & {} \frac{1}{N} \left| \sum _{j=1}^N y^{(\ell )}_m(j) e^{-i2\pi j k/N}\right| ^2, \quad \begin{array}{c} 1\le \ell \le p, \\ 1\le m \le n, \end{array} \end{aligned}$$

or, in terms of the standard FFT of the bootstrapped sample,

7 $$\begin{aligned} \varPhi _m^{(\ell )}(\nu _k) = \frac{1}{N}\left| \text{FFT}\big (y^{(\ell )}_m\big )_k\right| ^2. \end{aligned}$$

We arrange the periodograms in a stack of random matrices as indicated in Fig. 1, thus generating a three-dimensional array for each protocol and each subject.

Observe that the DC component, $\nu _0 = 0$, corresponds to the average power in each BR. The normalized PSDs of the BRs, defined as

8 $$\begin{aligned} \overline{\text{PSD}}_m(\nu _k) = \frac{\text{PSD}_m(\nu _k)}{\text{PSD}_m(0)}, \end{aligned}$$

represent the relative power density in the given frequency in each BR.

In this study, we are primarily interested in the distribution of the power among the $\alpha$- , $\beta$-, $\gamma$-, and $\theta$-spectral bands, corresponding to different brain rhythms. For each brain rhythm, we identify a characteristic frequency band,

9 $$\begin{aligned} I^i = \left[ \nu _{\text{min}}^i,\nu _{\text{max}}^i\right] ,\quad i \in \{\alpha ,\beta ,\gamma ,\theta \}, \end{aligned}$$

and define the sample matrix of bandwidth-specific integrated power vectors,

10 $$\begin{aligned} {{\mathsf {X}}}^{i} = \left[ \mathbf{x}^{(i,1)}, \mathbf{x}^{(i,2)},\ldots , \mathbf{x}^{(i,p)} \right] \in {{\mathbb {R}}}^{n\times p}, \end{aligned}$$

whose columns are sums of the scaled periodograms over the respective bandwidth, that is,

11 $$\begin{aligned} \mathbf{x}^{(i,\ell )}= & {} \left[ \begin{array}{c} x^{(i,\ell )}_1 \\ \vdots \\ x^{(i,\ell )}_n\end{array}\right] , \quad x^{(i,\ell )}_m = \frac{1}{\varPhi ^{(\ell )}_m(0)} \int _{\nu _{\text{min}}^i}^{\nu _{\text{max}}^i} \varPhi ^{(\ell )}_m(\nu ) d\nu \nonumber \\&\approx \sum _{ \nu _k\in I^i} \frac{\varPhi ^{(\ell )}_m(\nu _k)}{\varPhi ^{(\ell )}_m(0)} \varDelta \nu , \end{aligned}$$

where $i\in \{\alpha ,\beta ,\gamma ,\theta \}$. In addition to the four spectral bands, we also consider the total power vectors, or the DC components, defined as

12 $$\begin{aligned} \mathbf{x}^{(\text{DC},\ell )} = \left[ \begin{array}{c} x^{(\text{DC},\ell )}_1 \\ \vdots \\ x^{(\text{DC},\ell )}_n\end{array}\right] , \quad x^{(\text{DC},\ell )}_m = \varPhi ^{(\ell )}_m(0), \end{aligned}$$

and combine them into the matrix

13 $$\begin{aligned} {{\mathsf {X}}}^{\text{DC}} = \left[ \mathbf{x}^{(\text{DC},1)}, \mathbf{x}^{(\text{DC},2)}, \ldots , \mathbf{x}^{(\text{DC},p)}\right] . \end{aligned}$$

The spectral bands used in our analysis are defined in Table 1.

**Table 1:** Frequency bands used in the computation of the sample matrices ${{\mathsf {X}}}^{i}$

band	$\nu _{\text{min}}$	$\nu _{\text{max}}$	$\nu _{\text{peak}}$
$\theta$	$3.0\, (2.6) \,\text{Hz}$	$7.5\, (6.4)\,\text{Hz}$	$4.4 \,(3.7)\,\text{Hz}$
$\alpha$	$7.5\, (6.4)\,\text{Hz}$	$12.0\, (10.2)\,\text{Hz}$	$8.9\,(7.7)\,\text{Hz}$
$\beta$	$12.0\, (10.2)\,\text{Hz}$	$20.0\, (17.0) \,\text{Hz}$	$13.5\,(11.2)\,\text{Hz}$
$\gamma$	$25.0\, (21.3) \,\text{Hz}$	$40.0 \, (34.4)\,\text{Hz}$	-

The upper and lower bound of each band used in the calculation are $\nu _{\text{min}}$ and $\nu _{\text{max}}$, while $\nu _{\text{peak}}$ is the frequency at which the peak value occurs. In the $\gamma$ band, no unambiguous peak value was identified. The numbers outside the parentheses are the frequencies used for Subject 1, and those in the parentheses refer to Subject 2. The adjustment of the bands is based on the observed offset of the positions of the $\theta$-, $\alpha$-, and $\beta$-peaks

The process described above is carried out separately for the data sequences corresponding to the three brain states (rest, Vipassana, Samatha). In this manner, using the independently processed data from the different subjects, we construct independent samples of bandwidth-specific data matrices ${{\mathsf {X}}}^i_{\text{rest}}$, ${{\mathsf {X}}}^i_{\text{Samatha}}$, ${{\mathsf {X}}}^i_{\text{Vipassana}}$, where $i\in \{\text{DC},\alpha ,\beta ,\gamma ,\theta \}$.

### Activity Patterns and LDA

For the time being, for each spectral band and each subject, we consider the data corresponding to the three different protocols: Eyes closed (rest), focussed attention (Samatha) and open monitoring or mindfulness (Vipassana) meditation, generate the scaled integrated periodogram samples and collect them in the form of $n\times p$ matrices ${{\mathsf {X}}}^{(1)} = {{\mathsf {X}}}_{\text{rest}}$, ${{\mathsf {X}}}^{(2)} = {{\mathsf {X}}}_{\text{Samatha}}$, and $X^{(3)} = {{\mathsf {X}}}_{\text{Vipassana}}$. At this point we consider the following questions.

(i) Is it possible to separate the three brain states on the basis of the periodogram matrices?
(ii) Which BRs have more prominent roles in this separation?
(iii) Are there significant differences between different spectral bands of the brain activity?
(iv) Is the separation signature for each subject consistent across temporally separated sessions?
(v) Is the separation signature consistent across subjects?

To address these questions, we used LDA (Calvetti and Somersalo 2020), briefly summarized below. Historically, LDA was developed for separating two normally distributed samples (Fisher 1936), with an underlying homoscedasticity assumption. We point out that the LDA considered here is purely data driven and does not assume anything about the underlying distributions. We refer to standard data analysis and pattern recognition literature for details, see, e.g., (Calvetti and Somersalo 2020; Duda et al. 2012).

#### LDA

Linear discriminant analysis is a standard dimension reduction technique, suitable to analyze the clustering of annotated data. Given an annotated set of vectors in ${{\mathbb {R}}}^n$, organized as columns of distinct matrices according to their annotation to *k* clusters,

14 $$\begin{aligned} {{\mathsf {X}}}^{(1)}\in {{\mathbb {R}}}^{n\times p_1},\quad {{\mathsf {X}}}^{(2)}\in {{\mathbb {R}}}^{n\times p_2},\quad \cdots ,{{\mathsf {X}}}^{(k)}\in {{\mathbb {R}}}^{n\times p_k}, \end{aligned}$$

the LDA algorithm seeks a few vectors in ${{\mathbb {R}}}^n$ such that the orthogonal projections of each data set ${{\mathsf {X}}}^{(\ell )}$ onto these directions appears as a compact cluster of points, with minimal overlap. For completeness, below we briefly review the LDA algorithm for finding the separating directions.

For each matrix ${{\mathsf {X}}}^{(i)}$ whose columns are the vectors $\mathbf{x}^{(i,1)},\ldots , \mathbf{x}^{(i,p_i)}$ with identical annotation, referred to as a cluster, the corresponding $n\times n$ spread matrix is defined as

15 $$\begin{aligned} {{\mathsf {S}}}^{(i)} = ({{\mathsf {X}}}^{(i)} - \overline{\mathbf{x}}^{(i)}) ({{\mathsf {X}}}^{(i)} - \overline{\mathbf{x}}^{(i)})^{{\mathsf {T}}}, \quad \overline{\mathbf{x}}^{(i)} = \frac{1}{p_i}\sum _{\ell =1}^{p_i} \mathbf{x}^{(i,\ell )}, \end{aligned}$$

which is proportional to the empirical variance of the projected data. Given a vector $\mathbf{q}\in {{\mathbb {R}}}^n$, the scalar quantity $\mathbf{q}^{{\mathsf {T}}}{{\mathsf {S}}}^{(i)}{} \mathbf{q}$ is, by definition, the spread of the cluster around the cluster mean $\overline{\mathbf{x}}^{(i)}$ in the direction determined by $\mathbf{q}$. In statistical terms, the spread is proportional to the empirical variance of the projected data. To find the directions in which all the clusters have a small spread, we use the *within-cluster spread* matrix as the sum of the individual spread matrices,

16 $$\begin{aligned} {{\mathsf {S}}}_w = \sum _{i=1}^k {{\mathsf {S}}}^{(i)}, \end{aligned}$$

and observe that $\mathbf{q}^{{\mathsf {T}}}{{\mathsf {S}}}_w\mathbf{q}$ quantifies the aggregate spread of the data in the direction $\mathbf{q}$ after each cluster has been centered around its mean. Since we seek directions in which the clusters appear clearly apart from each others, to measure the separation we introduce the *between-cluster spread* matrix,

17 $$\begin{aligned} {{\mathsf {S}}}_b = \sum _{i = 1}^k p_i (\overline{\mathbf{x}}^{(i)} - \overline{\mathbf{x}})(\overline{\mathbf{x}}^{(\ell )} - \overline{\mathbf{x}})^{{\mathsf {T}}}, \end{aligned}$$

where $\overline{\mathbf{x}}\in {{\mathbb {R}}}^n$ is the mean of the aggregate data regardless of the annotation. The between-cluster mean can be thought of as a spread matrix obtained by replacing each data vector in a cluster by the corresponding cluster mean, thus ignoring the spread within the individual clusters.

Intuitively, the LDA algorithm seeks few directions such that the projected within-cluster spread in those directions is as small as possible, while the between-cluster spread is as large as possible. In other words, the LDA is looking for directions $\mathbf{q}\in {{\mathbb {R}}}^n$ for which the ratio

18 $$\begin{aligned} H(\mathbf{q}) = \frac{\mathbf{q}^{{\mathsf {T}}}{{\mathsf {S}}}_b \mathbf{q}}{\mathbf{q}^{{\mathsf {T}}}{{\mathsf {S}}}_w \mathbf{q}} \end{aligned}$$

is as large as possible. Observe that since the matrix ${{\mathsf {S}}}_w$ could be singular, to avoid division by zero we consider instead the modified ratio

19 $$\begin{aligned} H_\delta (\mathbf{q}) = \frac{\mathbf{q}^{{\mathsf {T}}}{{\mathsf {S}}}_b \mathbf{q}}{\mathbf{q}^{{\mathsf {T}}}({{\mathsf {S}}}_w +\delta {{\mathsf {I}}}) \mathbf{q}}, \end{aligned}$$

where $\delta >0$ is a small regularization parameter, and ${{\mathsf {I}}}$ denotes the $n\times n$ unit matrix.

The optimal projection direction is the vector $\mathbf{q}$ that maximizes the ratio $H_\delta (\mathbf{q})$. To find the optimal direction, we observe that $\mathbf{q}$ solves of the generalized eigenvalue problem of finding a pair $(\mathbf{q},\lambda )$ satisfying the equation

20 $$\begin{aligned} {{\mathsf {S}}}_b \mathbf{q} = \lambda ({{\mathsf {S}}}_w + \delta {{\mathsf {I}}}) \mathbf{q}, \end{aligned}$$

Because all generalized eigenvalues $\lambda$ are real and non-negative, and the generalized eigenvector corresponding to the largest generalized eigenvalue is also the maximizer of $H_\delta (\mathbf{q})$. Moreover, at most $k-1$ of the generalized eigenvalues are positive, thus there are at most $k-1$ separating directions. The LDA algorithm therefore seeks all the generalized eigenvectors that correspond to positive generalized eigenvalues. These vectors constitute the projection directions, and the dimensionality of the data can be reduced by representing the data points in terms of the LDA components, namely the orthogonal projections onto the separating directions. We point out that since, in general, the generalized eigenvectors are not mutually orthogonal, the LDA components may contain redundant information about the data. Orthogonalization of the LDA directions is possible, but we have observed (data not shown) that this usually leads to less clear cluster separation with little interpretative gain.

#### Optimal Window

When computing the periodograms through bootstrapping, the window length *N* in formula (4) plays an important role. Asymptotically, when shrinking the window to a single time slice all interdependency between time slices will be lost: from the physiological point of view, it is not plausible that snapshots over a window of one millisecond can reveal much of the current state of activity. On the other hand, increasing the window length eventually will result into a loss of independency of the bootstrap samples. Intuitively, the window should be long enough to contain details of biological relevance, but short enough to not blur the details. We therefore choose the window length based on the separation power of the LDA.

Given three data matrices ${{\mathsf {X}}}^{(j)}\in {{\mathbb {R}}}^{n\times p}$, $1\le j\le 3$, the LDA will find up to two directions corresponding to positive generalized eigenvalues, denoted by $\mathbf{q}^{(1)},\mathbf{q}^{(2)}\in {{\mathbb {R}}}^n$. Thus, the LDA provides the means to represent the data in just two dimensions, with LDA components of each cluster matrix ${{\mathsf {X}}}^{(j)}$ computed as

21 $$\begin{aligned} {{\mathsf {Z}}}^{(j)} = {{\mathsf {Q}}}^{{\mathsf {T}}}{{\mathsf {X}}}^{(j)}\in {{\mathbb {R}}}^{2\times p}, \quad {{\mathsf {Q}}}= \left[ \begin{array}{cc} \mathbf{q}^{(1)}&\mathbf{q}^{(2)}\end{array}\right] , \end{aligned}$$

where the vectors $\mathbf{q}^{(1)}, \mathbf{q}^{(2)}$ are of unit 2-norm. We define the *pairwise Bhattacharyya index* (Bhattacharyya 1943) to measure the mutual overlap as follows. Let $\varOmega$ denote a rectangle containing the projected data points in the plane determined by the LDA separating vectors $\mathbf{q}^{(1)}, \mathbf{q}^{(2)}$. We subdivide the rectangle $\varOmega$ into *N* bins, or pixels $\varOmega _\ell$, $1\le \ell \le N$, and denote by $\mathbf{h}({{\mathsf {X}}}^{(j)}) \in {{\mathbb {R}}}^N$ the histogram vector of the projected data with components

22 $$\begin{aligned} h_\ell ({{\mathsf {X}}}^{(j)}) = \frac{\# ({{\mathsf {Z}}}^{(j)}(1,k), {{\mathsf {Z}}}^{(j)}(2,k)) \in \varOmega _\ell }{p}, \end{aligned}$$

or the number of projected data points in $\varOmega _\ell$ normalized by the number of the data points. The pairwise Bhattacharyya index of the pair $({{\mathsf {X}}}^{(i)},{{\mathsf {X}}}^{(j)})$ is

23 $$\begin{aligned} \text{BI}({{\mathsf {X}}}^{(i)},{{\mathsf {X}}}^{(j)}) = \sum _{\ell =1}^N \sqrt{h_\ell ({{\mathsf {X}}}^{(i)}) h_\ell ({{\mathsf {X}}}^{(j)})}. \end{aligned}$$

Observe that if we interpret the histograms as probability densities, the quantity $1-\text{BI}(X^{(i)},X^{(j)})$ is the Hellinger distance between the respective probability measures. The overlap measure can be extended to $c>2$ classes by introducing the *mean pairwise Bhattacharyya index* (MPBI),

24 $$\begin{aligned} \text{MBPI}({{\mathsf {X}}}^{(1)},\ldots ,{{\mathsf {X}}}^{(c)}) = \frac{2}{c(c-1)}\sum _{i\ne j}^c \text{BI}({{\mathsf {X}}}^{(i)},{{\mathsf {X}}}^{(j)}). \end{aligned}$$

In this study, we use $c=3$ for the three brain states. Because the computation of the Bhattacharyya index depends on the binning of the rectangle $\varOmega$, we need to specify a criterion for the discretization. We use the Bayesian optimal binning algorithm (Knuth 2006) to set the binning density.

#### Separating Vector Analysis

LDA produces few (in the current setting two) separating directions in the *n*-dimensional data space, which in the present case is 165, the number of brain regions in the selected parcellation. As the components of the separating vectors refer directly to the relative power over the bandwidth in a brain region, the separating directions may be interpreted as indicators of the brain regions most significant for the separation between the brain states. Note that in the face recognition literature, similar use of LDA has been proposed to identify differentiating features between sets of faces, and the separating LDA directions are interpreted as face images, referred to as “Fisher faces” (Belhumeur et al. 1997).

Given the data matrices ${{\mathsf {X}}}^{(j)}$, $j=1,2,3$ containing the data of the three different brain states of a single meditator over a prescribed frequency band, let $\mathbf{q}^{(1)},\mathbf{q}^{(2)}\in {{\mathbb {R}}}^n$ denote the two LDA vectors. Each component $q^{(k)}_{\ell }$, $1 \le \ell \le n$ of the vector $\mathbf{q}^{(k)}$ refers to a brain region. If one of the *n* components of the separator vectors vanishes, i.e., $q^{(1)}_{\ell } = q^{(2)}_\ell = 0$ for some $\ell$, this implies that the components $z_k^{(j)} = (\mathbf{q}^{(k)})^{{\mathsf {T}}}{{\mathsf {X}}}^{(j)}$ are insensitive to the brain activity in the $\ell$th region. In that case, we may conclude that the $\ell$th brain region must play no role in the LDA separation, and therefore shows no particular specificity in the rest versus meditation practices. Conversely, if the $\ell$th component $q_{\ell }^{(k)}$ is large in one of the separator vectors, we may expect that the $\ell$th brain region has been identified as significant for the separation process. A similar reasoning is followed in the face recognition problem: A Fisher face reveals the facial features that are picked up by the LDA separation process, as opposed to generic features of little importance. Analogously, the separating vector significance hypothesis assumes that large components of the separating vectors identify brain regions that separate the meditative states from each other and from rest.

The LDA and the subsequent separating vector analysis that considers only the brain regions corresponding to large components of the separating vectors, while appealing, may be subject to a fallacy for at least two reasons. First, LDA is seeking directions that not only have optimal separation power, but along which the clusters appear as compact as possible. Therefore, a brain region may be identified as relevant for its effectiveness in the compactification of the clusters. Second, since the absolute activity levels differ from each other, it is possible that a strongly separating brain region with high absolute activation level may not require a large coefficient $q_{j,\ell }$ to still play a role. Summarizing, it is safer to assume that vanishing or almost-vanishing components correspond to insignificant regions than claiming that a large component is necessarily significant.

To mitigate the risk of over-interpretation, we identify the significant brain regions by means of an exclusion principle. More specifically, we apply a *sequential filtering process* that removes brain regions corresponding to relatively small separating vector components, and we repeat the LDA algorithm with the reduced data. If the cluster separation with the reduced data is still reasonably clear, this can be seen as an indication that the separating vector analysis is able to identify regions with separating power. The data reduction can be repeated several times, producing a nested set of index vectors, sequentially narrowing down the brain regions with separating power.

The LDA analysis and the subsequent separating vector analysis can be run also by restricting the data vectors to selected brain regions. Natural reduction schemes entail considering only the left or the right hemisphere, the cortical regions, or the internal structures.

## Results

The methodology described in the previous section is applied to data collected from two experienced meditators, referred to as “Subject 1” and “Subject 2”. Both subjects were male and right handed, Subject 1 was 40 years old with 27 years of meditation experience, and Subject 2 was 49 years old, with meditation history of 28 years. For both subjects, we select two sessions of each of the three protocols (rest, Samatha, and Vipassana), of the duration of approximately one minute, or 60 000 time slices, and referred to as “Set A” and “Set B” for both subjects. The sessions are temporally well separated from each other and preprocessed individually and independently to avoid correlations between preprocessing artifacts. For the description of the preprocessing, we refer to (Calvetti et al. 2019; Marzetti et al. 2014). Hence, the raw BR-ALI data comprise 6 data sets, for a total of $60,000$ vectors in ${{\mathbb {R}}}^{165}$ for each subject.

**Fig. 2:**
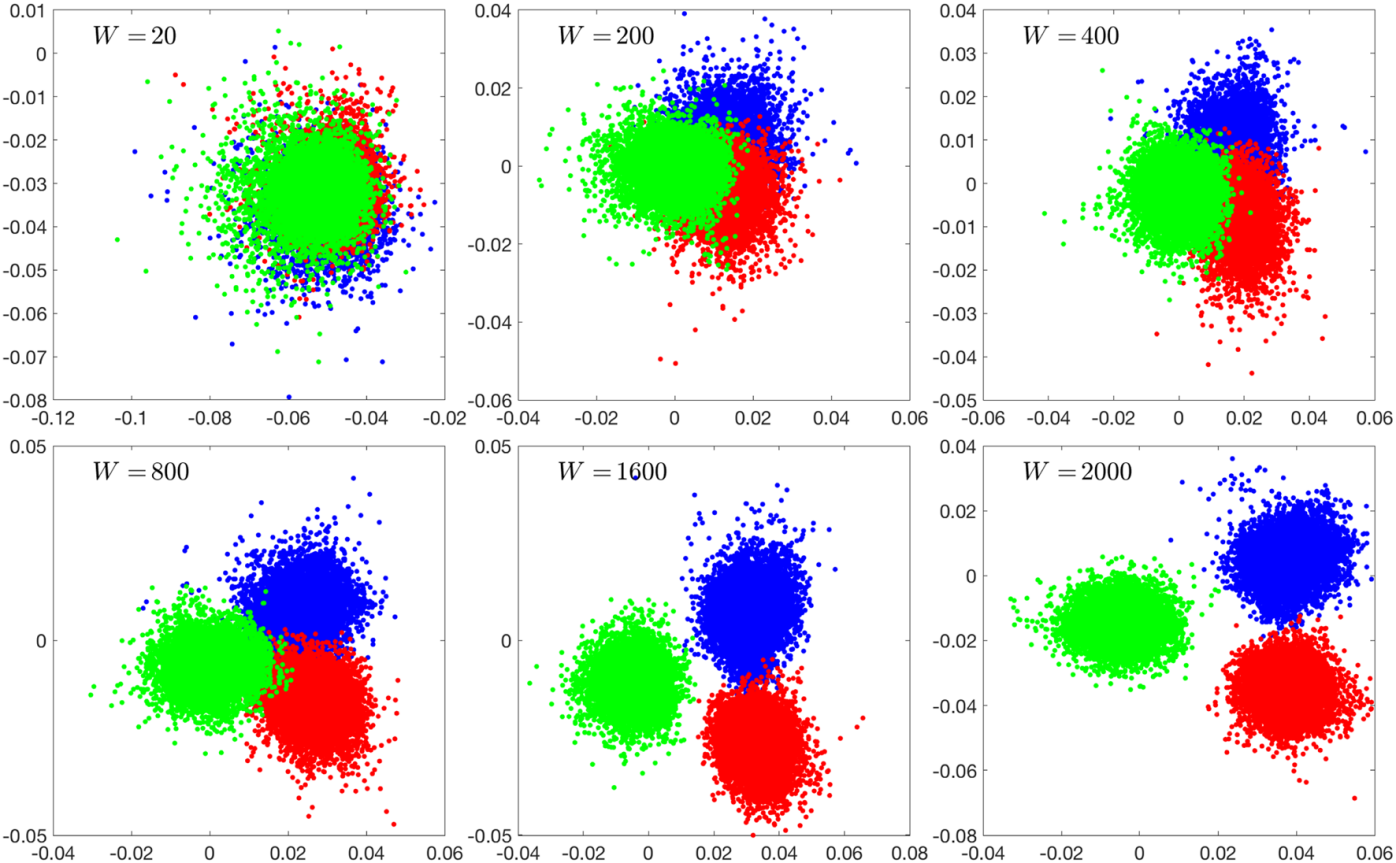
Scatter plots of the LDA components (first component (horizontal) vs. second component (vertical)) of the three meditation protocols of Subject 1 in the $\beta$ band, with different sampling window lengths used to compute the periodograms. The number of bootstrap samples is $10,000$ for each protocol. The resting state (or eyes closed) is in blue, Samatha in red, and Vipassana in green. The images, in lexicographical order, correspond to window lengths 20, 200, 400, 800, 1200 and 2000

**Fig. 3:**
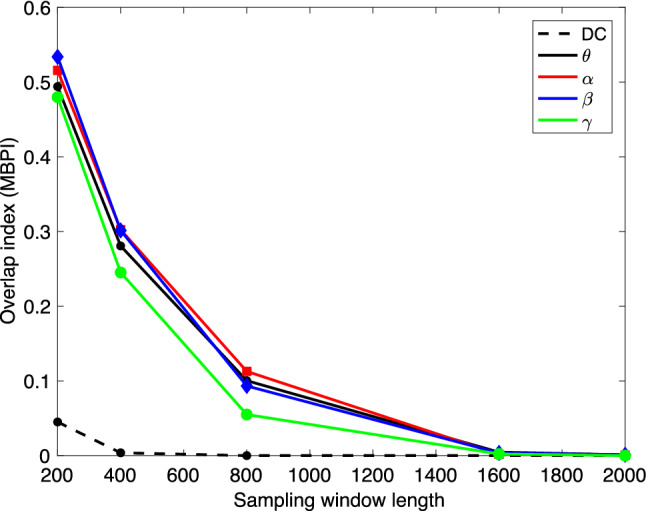
The $\text{MBPI}$ overlap index computed with different bootstrap window lengths

We start the LDA by considering the optimal windowing in the bootstrap process. To analyze the cluster separation by LDA, we select one data sequence corresponding to each of the three brain states (rest, Samatha, Vipassana) for Subject 1, and we compute the matrices ${{\mathsf {X}}}^{(j)}$, $j=1,2,3$ with different sampling window lengths *N* (see Eq. 4). Each matrix has $p = 10,000$ columns, and the sampling window length varies up to 2000. We then perform LDA for all selected spectral bands. Figure 2 shows the LDA projections of the $\beta$-band matrices of the three protocols for various window lengths, the corresponding scatter plots with other frequency bands being qualitatively very similar. As expected, short sampling windows do not allow a good separation, since for the method to recognize the characteristic spectral contents in the sample, and consequently to separate between the states, sufficiently long time series are needed. In Fig. 3, the pairwise overlap index is plotted for window lengths from 200 to 2000 for each one of the spectral bands. We see that, with the exception of the DC band, a window length of almost 2000 is necessary to have complete separation of the three states. Based on this observation, we set the window length to 2000.

**Fig. 4:**
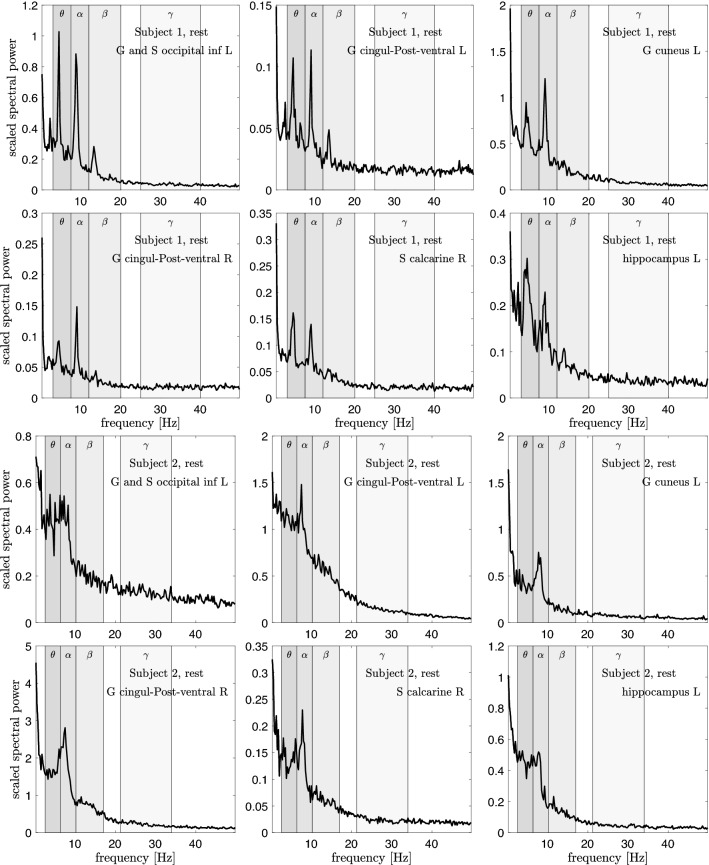
Six scaled power spectral densities corresponding to eyes closed resting state of Subject 1 (two top rows) and Subject 2 (two bottom rows). The scaled PSD values have been scaled by a factor of 100. Observe that each PSD is scaled differently. The peak values for Subject 2 appear at a frequency lower than those of Subject 1, and the frequency bands are adjusted accordingly, see Table 1

**Fig. 5:**
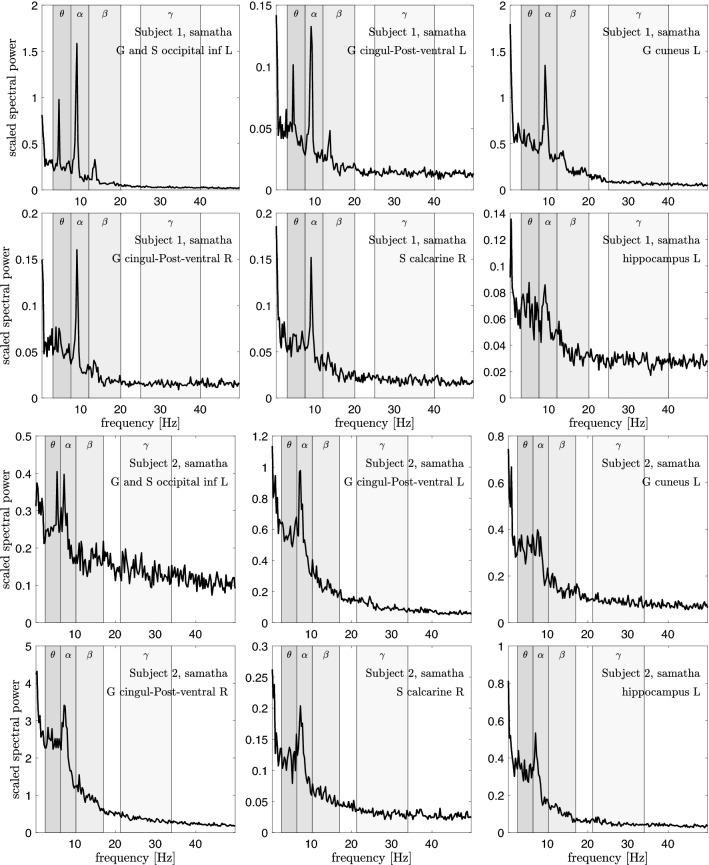
Six scaled power spectral densities corresponding to focused attention (Samatha) meditation state of Subject 1 (two top rows) and Subject 2 (two bottom rows). The scaled PSD values have been scaled by a factor of 100. Observe that each PSD is scaled differently. The peak values for Subject 2 appear at a frequency lower than those of Subject 1, and the frequency bands are adjusted accordingly, see Table 1

**Fig. 6:**
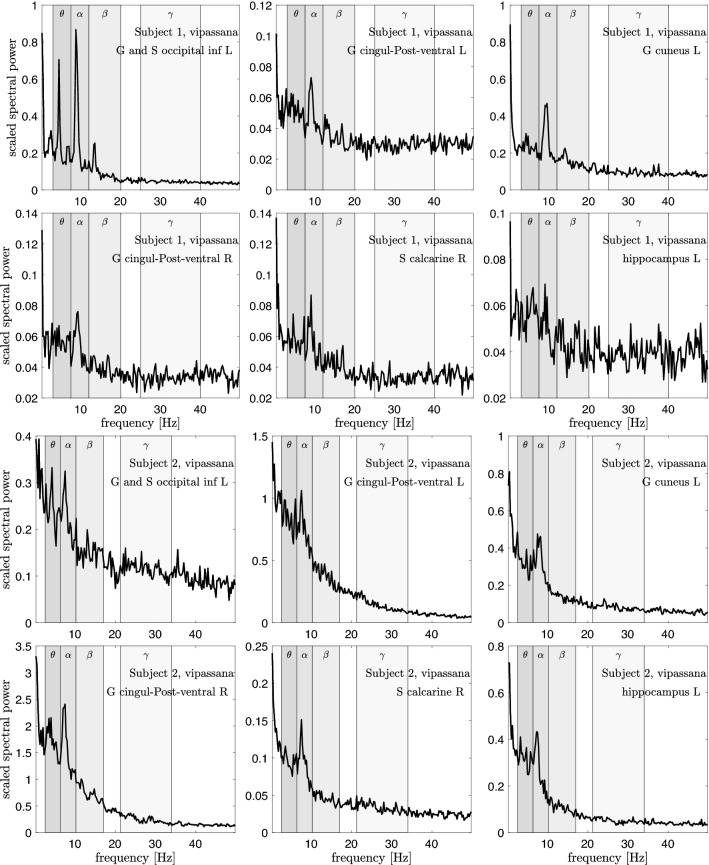
Six scaled power spectral densities corresponding to open monitoring (Vipassana) meditation state of Subject 1 (two top rows) and Subject 2 (two bottom rows). The scaled PSD values have been scaled by a factor of 100. Observe that each PSD is scaled differently. The peak values for Subject 2 appear at a frequency lower than those of Subject 1, and the frequency bands are adjusted accordingly, see Table 1

Figures 4, 5 and 6 show the scaled power spectral densities of six selected brain regions. The densities are calculated using a bootstrap sampling window of length $N=2000$, and the number of samples is $p=10,000$ for each brain state. The regions included in these plots are selected by visual inspection to represent pronounced spectral spikes, in particular the $\alpha$ spike. The spikes of Subject 1 are more outstanding, while a clear $\beta$ spike is not easy to identify in Subject 2. This may be due to the different quality of the measured data for the two subjects. Importantly, a systematic analysis of the PSDs demonstrates that the $\theta$-, $\alpha$-, and $\beta$-spikes of Subject 2 occur at lower frequencies than those of Subject 1, the ratio of the frequencies being approximately 0.85. Based on this offset, we adjust the frequency bands by the same factor.

**Fig. 7:**
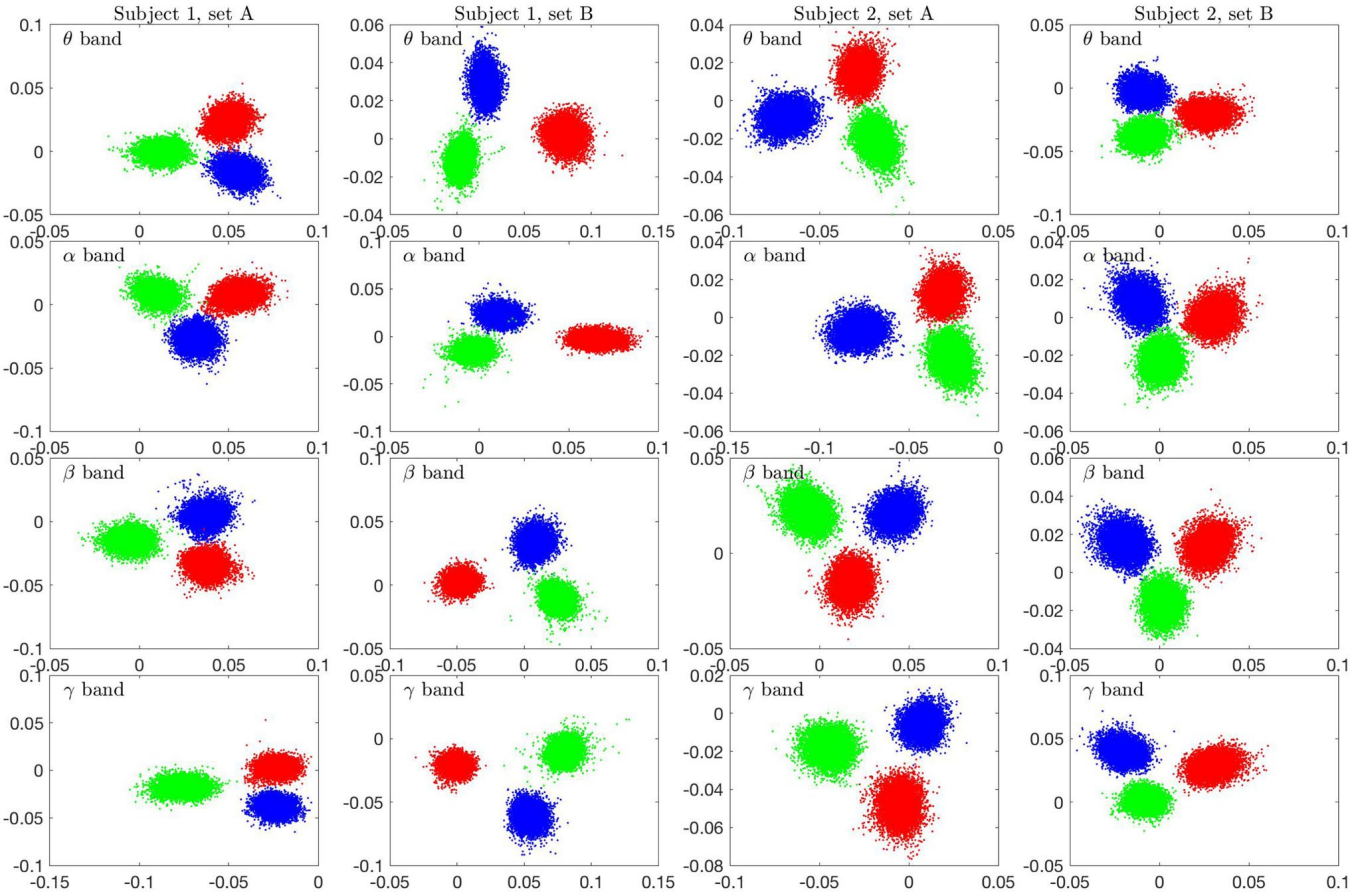
Scatter plots of the LDA components (first component (horizontal) vs. second component (vertical)) corresponding to the four independent data sets (two sets per subject), and for the four frequency bands. The bootstrap window is $W = 2,000$. Blue dots correspond to eyes closed resting state, red dots to Samatha, and green dots to Vipassana

Figure 7 shows the LDA scatterplots of the four data sets, sets A and B for both subjects, and for each of the four frequency bands. The DC component has not been included because the arbitrary scaling of the data may affect the separation. Furthermore, the four frequency bands shown here are scaled by the DC component, to remove the possible separating effect due the scaling. We see that the LDA cluster separation is consistent across the different data sets and the frequency bands.

Next we examine the LDA separating vectors to identify the brain regions with the most significant role in the LDA separation. To identify the brain regions that in the light of the separating vectors play the key role in separating the states, as explained in "Separating Vector Analysis" section, we run the following sequential filtering algorithm. Given the data set of three brain states, we first perform the LDA to find two separating vectors $\mathbf{q}^{(1)}$ and $\mathbf{q}^{(2)}$ for each frequency band. Next, we reduce the dimensionality of the data by discarding the rows whose indices $\ell$ satisfy

25 $$\begin{aligned} |q_{\ell }^{(1)}|<0.1 \max _{1\le j\le n} |q_{j}^{(1)}| \quad \text{ and } \quad |q_{\ell }^{(2)}|<0.1 \max _{1\le j\le n} |q_{j}^{(2)}|. \end{aligned}$$

**Fig. 8:**
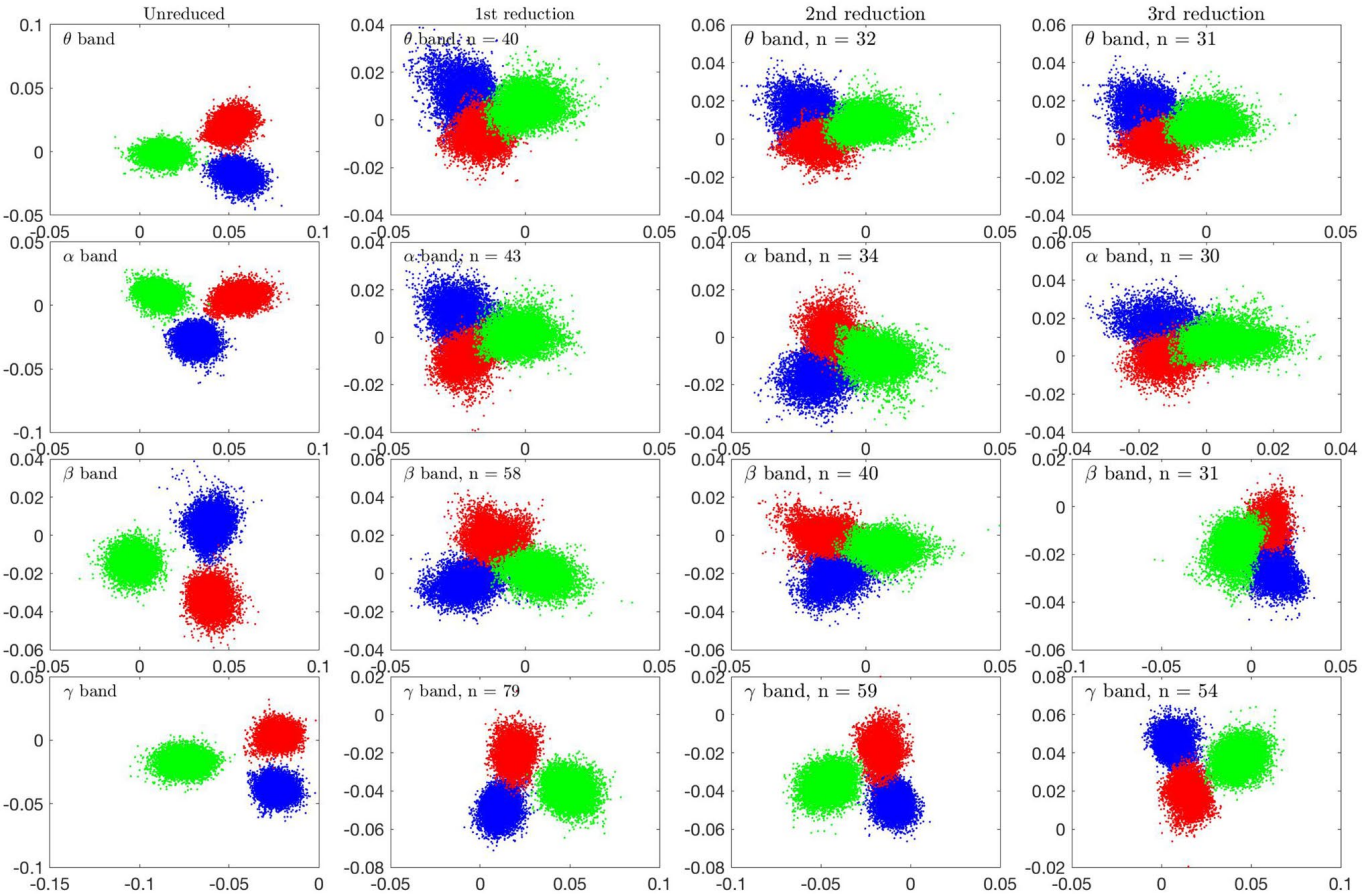
LDA separation combined with LDA-guided model reduction using the data of Subject 1. In the left column, the scatter plots of the LDA components of different spectral bands are shown (first component (horizontal) vs. second component (vertical)). Blue dots correspond to eyes closed resting state, red dots to Samatha, and green dots to Vipassana. In the second column on the left, the brain regions corresponding to components in the LDA separating vectors with less than 10% of the maximum component are discarded, and the LDA analysis is performed with the reduced model. The process is continued similarly, removing progressively the brain regions with a low component in the separating vector. In each figure, the number *n* of retained components are indicated

In other words, a brain region is deemed as a significant separator if the amplitude of the corresponding component in at least one of the separating vectors is more than 10% of the maximum amplitude of the components of that vector. This process leads to different model reduction for each frequency band. We then perform LDA with each of the reduced data matrices, and repeat the process, retaining only a subset of the brain regions considered in the previous round. In Fig. 8, the scatter plots of the LDA projections after one, two and three model reductions are shown, and the number of retained brain regions (index *n* in the figures) is indicated. The plots show that by discarding brain regions, the separation of the clusters deteriorates, as one would expect, however the separation is not lost even after three reduction steps. While the conclusions are not quantitatively definitive, the result can cautiously be interpreted to support the separating vector significance hypothesis: The activity in brain regions corresponding to small components of the separating vectors are insignificant for separation of the states. Moreover, if the components with the largest amplitudes would not correspond to separating brain activity, one would expect a rapid loss of the separation.

Assuming that the separating vector significance hypothesis is valid, we then turn to the key question of which brain regions stand out in the separating vectors. To make the analysis statistically more meaningful, we consider all possible mixtures of the three brain states for both subjects. Having two independent data sets (A and B) of each state, we may choose the eyes closed resting state in two ways, the Samatha in two ways, and the Vipassana in two ways, leading to eight different combinations. We perform the LDA for each of the eight combinations, and identify the components whose amplitude is at least 10% of the corresponding maximum amplitude of that vector. Having thus identified the most prominent components, we compute a tally of the brain regions that were selected at least once.

**Fig. 9:**
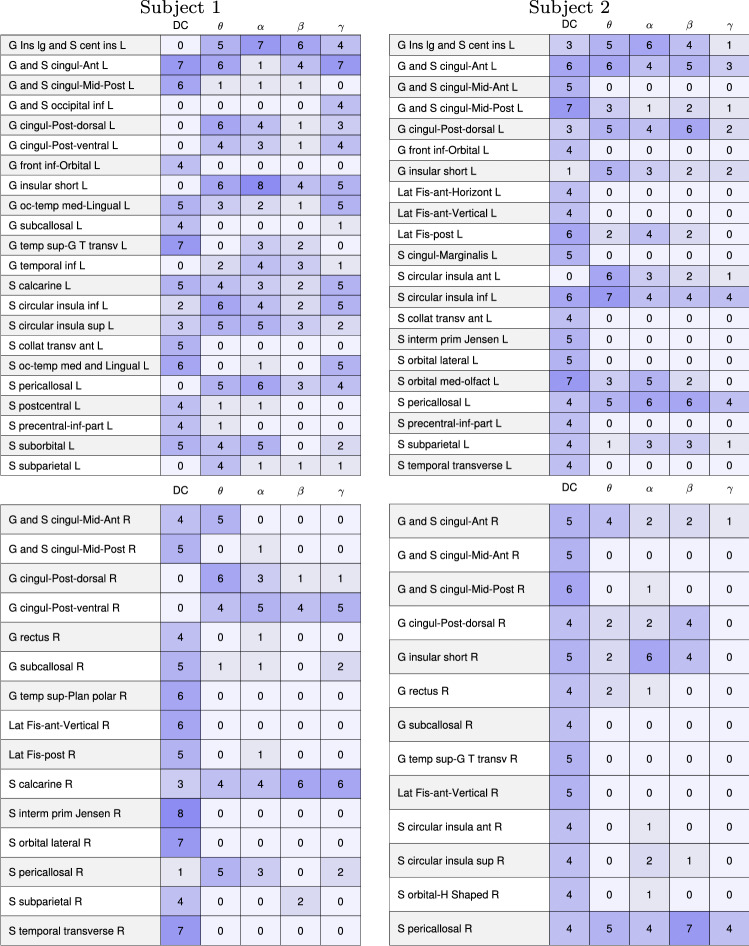
Tally of the cortical left (L) and right (R) brain regions of Subject 1 (left) and Subject 2 (right) that are identified as having at least 10% amplitude of the maximum amplitude of the separating LDA vectors (see Table 2 in the Appendix for long names of regions). With eight possible combinations of the three meditative states, the tally is at most 8. Only the brain regions that were identified at least once are shown. The shade of the blue is visualizing the tally results

**Fig. 10:**
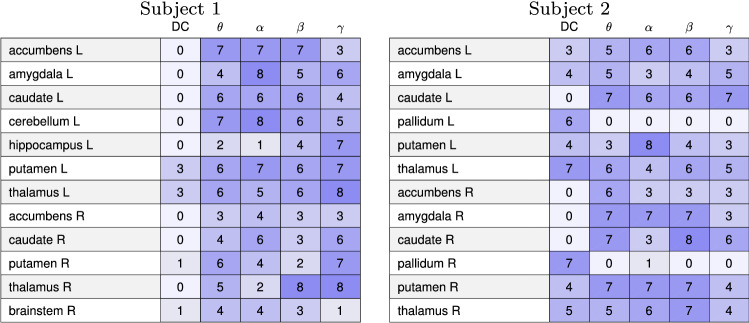
Tally of the internal left (L) and right (R) brain regions of Subject 1 (left) and Subject 2 (right) that are identified as having at least 10% amplitude of the maximum amplitude of the separating LDA vectors. With eight possible combinations of the three meditative states, the tally is at most 8. Only the brain regions that were identified at least once are shown. The shade of the blue is visualizing the tally results

Although the overall DC power shows clear separation capabilities, as reported in the results, the risk of having significant separation of the states because of arbitrary scaling of the reconstructions deems the DC separation as potentially less reliable. Our scaling of the periodograms by the DC component makes the frequency band analysis less prone to effects of different preprocessing of each session.

The tallies of the cortical brain regions of both subjects that are identified as having at least 10% amplitude of the maximum amplitude of the separating LDA vectors are reported in Fig. 9, while Fig. 10 reports the results relative to the internal brain structures. With eight possible combinations of the three meditative states, the tally is at most 8.

**Fig. 11:**
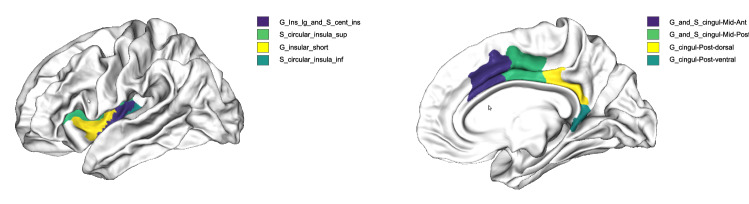
The left and right hemisphere cortical brain regions that are found to play a significant role in the separation of meditative and resting brain states

**Fig. 12:**
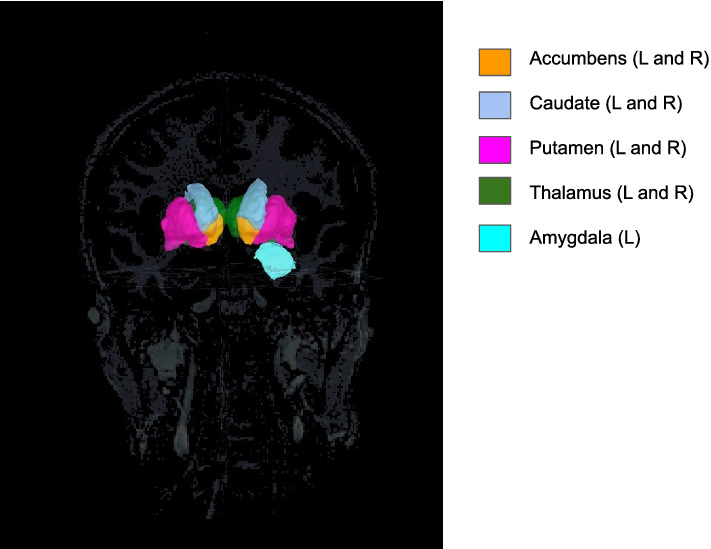
The internal brain structures that are found to play a significant role in the separation of meditative and resting brain states

The cortical regions consistently associated with the support of the separating vectors for the three brain states are located in the proximity of the internal structures, with the left hemisphere more conspicuously represented than the right one. The separator vectors for both subjects show a large component in all four spectral bands, corresponding to the anterior part of the cingulate gyrus and sulcus (ACC), the posterior-dorsal part of the cingulate gyrus (dPCC) and the pericallosal sulcus in both hemisphere. Furthermore, the insular cortex is prominently present in the separating brain regions. Figures 11 and 12 summarize visually the main discriminant regions.

## Discussion

To put the results presented in the previous section into proper context, we briefly review some of the findings reported in the literature. For the most part, the understanding of how meditation affects the brain is not based on functional imaging, and the connection with our findings is therefore not straightforward.

### Earlier work

A significant amount of meditation related brain data deals with anatomical changes presumably due to medium to long term (over eight weeks) meditation practice. Consistent anatomical changes in specific brain regions of trained meditators have been reported in the literature. We refer to a recent meta-analysis done by Pernet et al. (2021), summarizing the results in 25 MRI-based studies of brains of meditators. A significant number of the studies show changes in the gray matter thickness, most notably in the insulae and the anterior cingulate and the paracingulate gyri. In addition to the anatomical changes in the brain, recently there has been a sustained effort to quantify possible changes in brain functions associated with meditation practices, Fox et al. (2016), in an effort to better understand the mechanism behind meditation and to explore interventional meditation training in clinical settings. Dynamic changes in brain functions during meditation are less well understood than morphological ones, partly because of the difficulty of collecting data. While BOLD fMRI may reveal changes in cerebral blood flow, the data collection process is a distracting factor that cannot be neglected. Much less disruptive data collection modalities include EEG and to some extent MEG. In the literature there are studies where the MEG data during meditation practice has been investigated from the point of view of activation patterns and connectivity (Marzetti et al. 2014; Wong et al. 2015; Aftanas and Golocheikine 2001; Yamamoto et al. 2006; Kerr et al. 2011; Lee et al. 2018; Kiebel et al. 2005). Other studies are looking at MEG meditation data for psychological and cognitive interpretation (Dor-Ziderman et al. 2013; Sasaki et al. 1996), and to understand changes in the sensation of pain (Kakigi et al. 2005). In the activation studies, the starting point is often the fMRI based functional understanding of activation patterns such as the default mode network (DMN). A number of neuroimaging studies found that meditation practices induce significant changes in the emotion regulating areas of the brain, particularly the insula, the amygdala and the basal ganglia regions, that align with the self-reported state of well being associated with meditation practices.

The main motivation for our analysis is to find out whether the effects of meditation, either long term or during the practice, can be characterized by changes in neural oscillations activated in the different brain regions. Several papers have addressed this question, focusing on different wave bands. Based on EEG measurements, the $\alpha$ power has been reported higher in Vipassana than during rest mainly at frontal and fronto-central electrodes (Braboszcz et al. 2017). While $\alpha$ activity is not considered a hallmark of meditative states, it is generally associated with redirection of attention to internal objects, and it has been hypothesized (Posner 2018) that an increase in the $\alpha$ spectral power plays a role in thalamo-cortical sensory transmission, hence relating to functional inhibition, and to the suppression of irrelevant input, tantamount to increasing the gating of distracting stimuli.

Although the significance of the increase in the power of $\gamma$ waves (20–100 Hz) is not fully understood, these brain rhythms are correlated with working memory and attention. An increase in $\gamma$ power recorded by EEG has been related, although not in a definitive manner, to ongoing stream and contents of consciousness, and visual representation (Braboszcz et al. 2017). Generally, $\gamma$ power over parieto-occipital electrodes is higher in meditators than control, and findings support the hypothesis that training in mindfulness meditation increases the $\gamma$ power proportionally to experience (Cahn et al. 2010). An EEG-based study by Lutz et al. (2004) reported increase in the $\gamma$ band in fronto-lateral and posterior electrodes, while (Cahn and Polich 2009) found increased $\gamma$ in parieto-occipital electrode during Vipassana practice. Another study (Braboszcz et al. 2017) that compared three styles of meditation found the increase in the higher (60–110 Hz) $\gamma$ band in electrodes in parieto-occipital area in meditators over control to correlate positively with the experience of the meditators.

The potential of mindfulness meditation as a clinical tool for depression is a topic of active investigation. Yang et al. (2016, 2018), using resting state fMRI data to investigate the structural and functional changes in the brain of novices following a 40-day training in mindfulness meditation (Vipassana), found a significant thickening of the precuneus region and a decrease in $\alpha$ amplitude. These changes are suggestive of resting-state network changes following meditation training, and correlate with reduction in depression scores. A review of mindfulness meditation in substance abuse can be found in Zgierska et al. (2009).

In an effort to correlate changes in brain connectivity and depression states to determine whether it is possible to establish a priori if a subject is likely to benefit from meditation, a recent study (Doborjeh et al. 2019) applied a spiking neural network model to EEG measurements in different wave bands before and after a six week mindfulness meditation training period for non-depressed (ND), depressed before but not after training (D+), and depressed before and after training (D−) individuals. The findings pointed to an increase in spatio-temporal connectivity over the frontal, centro-parietal and occipito-parietal areas in the ND group after training, with less significant increases for the D+ group, and minimal changes in the D- group.

In an effort to find coherence between the findings of different groups on which frequency bands that may be altered during a meditation session or following meditation training, Lomas et al. (2015), after a systematic review of 56 papers, for a total of 1715 subjects, reported an agreement that $\alpha$ and $\theta$ powers are higher during mindfulness meditation than at rest, while there was no overall consistency in the reported changes in $\beta$, $\gamma$ and $\delta$ power. Some of the papers, however, found increase in $\theta$, and to a lesser extent in $\alpha$ power in anterior cingulate cortex (ACC) and adjacent prefrontal cortex.

### Our Findings

#### Methodology

The MEG inverse problem was solved by using the IAS algorithm (Calvetti et al. 2015) that was developed in particular for identifying deep brain activity. The algorithm is numerically efficient, and its performance in identifying active brain regions using Bayes factors was assessed in Calvetti et al. (2019) with favorable comparison to some popular inversion methods such as wMNE (Lin et al. 2006), dSPM (Dale et al. 2000) and sLORETA (Pascual-Marqui 1999). The LDA was chosen as a method of dimension reduction due to its sensitivity of differences in annotated data clusters as well as the high interpretability in terms of identifying separating attributes (Calvetti and Somersalo 2020). The spectral analysis of the spatially resolved data with windowing and bootstrapping to process the data is, to the best of our knowledge, a novel contribution to analyzing time series data in this context. In Hornero et al. (2008), LDA was applied to spectral data of the aggregated background signal, and dissimilarity measures of datasets, combined with different clustering algorithms were discussed in Guggenmos et al. (2018), however, without the steps of resolving the MEG inverse problem and spectral analysis, which are crucial for the interpretability of the results.

In the following, we highlight some of the brain regions that appear to play a significant role in the separation of the activities, and present a short review of previous literature that supports the findings.

#### Anterior and Posterior Cingulate Cortex (ACC and PCC)

The analysis identified areas in the anterior and posterior cingulate cortex as loci of separating activity. The cingulate cortex is an integral part of the limbic system. The limbic system is involved with the formation and the processing of emotions, and with learning and memory processes. Changes in the posterior cingulate cortex related to mindfulness meditation have been the topic of several studies (Brewer and Garrison 2014).

In Brewer et al. (2011a), the focus is to understand whether meditation changes the connectivity of the PCC as part of the DMN. In light of the observation that the non self-referential nature of mindfulness meditation is likely to decrease PCC activity (Garrison et al. 2013; Quin and Northoff 2011; Breczynski-Lewis et al. 2007), it is not surprising that this BR plays a role in the separation, although our investigation does not specify whether the open monitoring meditation (Vipassana) increases or decreases activation in the PCC. The ACC, also appearing as one of the BRs playing a role in separating the three brain states, is generally believed to contribute to cognitive control and emotional regulation.

Increased gray matter density in the left hippocampus, the PCC, the temporo-parietal junction, and the cerebellum, all regions involved in learning, memory processes, emotion regulation self-referential and perspective processes, has been reported (Hölzel et al. 2011) after eight weeks of mindfulness meditation training. Professional meditation activity is a long and demanding path, therefore a question that has received attention in the literature, in the context of using mindfulness meditation as an interventional clinical tool, is whether a short-term meditation training can change the brain structure and functions (Kozaka et al. 2018). Higher fractional anisotropy around the ACC, measured via diffusion tensor imaging after two weeks of mindful meditation, reported in Tang et al. (2009), is considered as a proxy for increased white matter pathways. Changes in myelination and axon density after four weeks of mindful meditation have been reported in Tang et al. (2010).

Dunlop et al. (2017), who examined mindfulness meditation in the context of major depression disorders, reported functional connectivity of the sub-callousal cingulate cortex (SCC) with the left anterior ventrolateral prefrontal cortex/insular dorsal midbrain, and the left ventromedial prefrontal cortex. Major depression is associated with relative hyperactivity in the limbic brain regions, (amygdala, insula and SCC), and hypoactivity in the dorsolateral prefrontal cortex. Moreover, elevated metabolism in SCC appeared to be a predictor of poor outcome of antidepressants.

A number of functional connectivity/neuroimaging related studies focus on changes in the DMN, which is known to deactivate during the performance of tasks. A review of the literature on functional and structural neuroimaging studies of the neural processes associated with mindfulness meditation (Marchand 2014) provides compelling evidence of the impact of meditation on the medial cortex and associated DMN, the insula and the amygdala, and some evidence of effects on the basal ganglia and latero-frontal regions. The role of meditation in the regulation of brain networks continues to be actively studied (Jang et al. 2011). Changes in the DMN, and in particular on the PCC connectivity, associated with mindfulness meditation practice have been addressed in several studies, motivated by the observation that higher DMN activation correlates with attention lapse and anxiety. Reduction in DMN activity during the practice of mindfulness meditation was first described in Brewer et al. (2011a). Decrease in PCC activity during mindfulness meditation is quite plausible, as this is a non self-referential task, and some studies (Quin and Northoff 2011; Breczynski-Lewis et al. 2007) show that trained monks have lower PCC activity than novice meditators. Furthermore, while investigating a possible role of mindfulness meditation in smoke cessation and other behavioral interventions, Brewer and Garrison (2014), Brewer et al. (2011b) identified the DMN, and in particular the PCC, as some of the regions primarily affected by meditation. The analysis of MEG recording in a cohort of experienced meditators (Marzetti et al. 2014) found the coupling in the $\alpha$ frequency band of the PCC with different brain regions to change according to the type of meditation practice. A difference in the level of $\alpha$ activity between experienced and novice meditators was reported in Wong et al. (2015).

Tang et al. (2019) found increase in $\theta$ power in frontal midline (FM) EEG electrode following mindfulness meditation and hypothesized that the increase may result in a proliferation of oligodendrocytes, hence increased myelination and in turn improved the connectivity between the ACC and the limbic areas; see also (Posner 2018; Posner et al. 2014). Earlier on, an increased $\theta$ activity was found to be positively correlated with glucose metabolism in the ACC (Pizzagalli et al. 2013). EEG dynamics in Vipassana meditators (Kakumanu et al. 2018) shows increase in $\delta$ (1–4Hz) and low $\gamma$ (30–40Hz) power at baseline, and increase in $\alpha$ and low $\gamma$ power during mindfulness practice.

#### Insular Cortex

The insular cortex (left for Subject 1, and both sides for Subject 2, see Fig. 11 for the main discriminant regions^1^) seems to have a significant prominence in many frequency bands. The insular cortex is credited with playing a significant role in a range of processes, including feelings and emotions, bodily- and self-awareness, decision making, sensory processing, and social functions such as empathy (Gogolla 2017). The central role of the insular cortex in meditation practice has been implicated in Luders et al. (2012) where an increase in cortical gyrification in meditators was observed. A cortical thickening of the insular cortex of long term meditators, observed in Engen et al. (2018), was taken as an indication that this brain region has a central role in meditation. The importance of the insular cortex in the context of meditation was highlighted in Dunlop et al. (2017) also. In Mooneyham et al. (2017), the authors report increases in the left hemisphere posterior insula and in its functional connectivity with the middle and superior left temporal gyrus, and the right ventrolateral prefrontal cortex after a six weeks’ training bootcamp in mindfulness meditation. These brain regions comprise a structurally connected network involved in early auditory perception, attention to changes in sound, and the analysis of sound fluctuations. In Kilpatrick et al. (2011), meditators were found to have increased connectivity between the prefrontal cortex (PFC) network and the posterior insula.

#### Internal Structures

In spite of their relative small volume and distance from the sensors, the internal structures have the lion’s share among the attributes most relevant for separating the three brain states. More specifically, the left and right accumbens, the caudate and the putamen nuclei, together with the left and right thalamus, and the left amygdala are significantly represented in the support of the separating directions for both subjects (see Fig. 12).^2^ The putative roles of these regions in the regulation of activities and emotions is in line with the reported different mental states associated with the three protocols. The nucleus accumbens and the caudate are among the internal structures consistently represented in the vectors separating the three different states for both subjects in all frequency bands. The accumbens, a region in the ventral striatum, has been extensively studied over the last half century, and because of its involvement with the reward circuit, it is often referred to as the pleasure center of the brain. In a recent comprehensive review of the role of the nucleus accumbens (Floresco 2015), this brain region has been characterized as an aid to obtain motivational relevant goals by promoting behaviors leading to their attainment. This aligns well with the state of well being usually associated with Vipassana, and the steps needed to reach it.

Another striatum region, with a significant role in separating brain states for both subjects, is the caudate nucleus, also related to the reward system and part of the cortico-ganglia-thalamic group. The caudate nucleus, typically associated with a number of clinical conditions related to movement, including Parkinson and Huntington diseases, has a significant role in cognitive functions associated with learning and memory. A retrospective review of the literature on the different roles of the caudate and accumbens nuclei (Grahn et al. 2009) concluded that the caudate contributes to learning and memory by overseeing processing that are fundamental to all tasks involved in goal-directed actions. In light of the strong goal-directed component in Samatha and Vipassana meditation, it is not surprising that the level of activation of the caudate nucleus acts is one of the factors implicitly used by LDA to cluster and separate the different states.

The activity in all frequency bands in the putamen nucleus which together with the caudate nucleus forms the dorsal striatum, the region over and to the side of the limbic system, also consistently appears among the attributes characterizing brain changes during meditation. The putamen, whose anatomical structure is very similar to that of the caudate, while mostly associated with preparation of limb movements, has been shown to have a role in category learning (Ell et al. 2006) also.

The amygdalae, also part of the basal ganglia, are credited with a role in emotions and behavior, especially in response to fear and anxiety. Electrical stimulation of the right amygdala has been shown to elicit negative emotions, in particular anxiety and sadness, while electrical stimulation of the left amygdala has produced either positive or negative emotions. The consistent presence of the left amygdala among the separating regions suggests changes in its activation at all frequency band levels during meditation practices. This is in line with literature reporting structural changes of the amygdala in relation to stress reduction (Hölzel et al. 2010), and reduction of the volume of the right amygdala related to meditation and yoga practices (Gotink et al. 2018). Effects of mindfulness meditation on the amygdala are reported also in Marchand (2014). The presence of the left and right thalamus among the BRs represented in the separating vectors for all frequency bands reinforces the hypothesis that meditation induced changes occur mostly in deeper brain regions. Anatomical investigations found increase in gray matter volume of the left hippocampus, the thalamus and the caudate among yoga practitioners versus control (Gothe et al. 2018). A recent MRI-based study (Kral et al. 2018) found the impact of meditation practice in the form of decreased reactivity of the amygdala in response to positive pictures and increased connectivity of the amygdala with ventromedial prefrontal cortex, a region implicated in emotion regulation and in the processing of self-referential stimuli (Northoff and Bermpohl 2004).

A retrospective study (Deai et al. 2015) to determine potential physiological and structural changes of yoga and meditation on brain waves reported an increase in gray matter and the amygdala, and frontal cortex activation, raising the possibility of using these practices in clinical treatment and to promote successful aging (Sperduti et al. 2017). Other studies corroborate the increase in gray matter volume of the left hippocampus, the thalamus and the caudate among yoga practitioners versus controls (Gothe et al. 2018). Changes in hippocampal anatomy in long-term meditators, in the form of a larger volume, where reported in Luders et al. (2012).

The MRI-bases study on the role of the basal ganglia in Gard et al. (2015) takes a hypothesis-free approach, by looking at all brain regions instead of limiting the attention to subnetworks. The data, consisting of time series measurements at resting states of yoga practitioners, Vipassana practitioners and control subjects, found that, in Vipassana and yoga practitioners, the caudate is more connected to other brain regions than in the control group, in line with the increased caudate activity. The fMRI-based study in Baerentsen et al. (2010) found increased putamen activity at the onset of meditation and increased caudate activity during sustained meditation; another study (Sperduti et al. 2012) also proposes a model of meditative state involving the putamen and the caudate. The involvement of the caudate in mindfulness meditation confirms the related caudal connectivity to cognition, emotion, action and perception (Robinson et al. 2012), and is in line with goal-oriented learning being meditated by the caudate, and habitual learning by the putamen (Braunlich and Seger 2013), and stress having been defined as a shift from goal orient to habitual behavior (Schwabe and Wolf 2009) .

## Conclusions

This study aims at investigating whether it is possible to quantify dynamic changes in brain activity during meditation practices versus rest based on the MEG data collected during meditation sessions. The work focuses on four frequency bands, $\alpha$-, $\beta$-, $\gamma$-, and $\theta$-bands, and uses LDA to identify those brain regions in which the activity undergoes changes significant enough to give rise to an identifiable separation of the bootstrapped periodograms computed from the spatially resolved sequence of brain activity. The results indicate that the internal structures, constituting the core of the limbic system, play a fundamental role in the separation. In addition, certain cortical areas, most notably the insula and the cingulate cortex, stand out as possible areas in which the activity differs from resting state activity during the practice. The findings are in line with existing literature that for the most part focuses on anatomo-morphological changes in the brain trained by meditation practice.

## Data Availability

MEG data used in this study was recorded at the Institute of Advanced Biomedical Technologies (ITAB), University ’G. d’Annunzio’ of Chieti (Italy) and have also been used in Marzetti et al. (2014). The data set was kindly made available to us by V. Pizzella, and we are not allowed to share them. Original MEG recordings should be asked to V. Pizzella. Post-processed data and reconstructed activity maps are available upon request.

## Appendix

See Table 2

**Table 2:** List of anatomical parcellation in the Destrieux atlas (Destrieux et al. 2010)

Short name	Long name
G $\_$and$\_$S$\_$occipital $\_$inf	Inferior occipital gyrus and sulcus
G$\_$and$\_$S$\_$cingul$\_$Ant	Anterior part of the cingulate gyrus and sulcus
G$\_$and$\_$S$\_$cingul-Mid-Ant	Middle-anterior part of the cingulate gyrus and sulcus
G$\_$and$\_$S$\_$cingul-Mid-Post	Middle-posterior part of the cingulate gyrus and sulcus
G$\_$cingul-Post-dorsal	Posterior-dorsal part of the cingulate gyrus
G$\_$cingul-Post-ventral	Posterior-ventral part of the cingulate gyrus
G$\_$cuneus	Cuneus
G$\_$front$\_$inf-Opercular	Opercular part of the inferior frontal gyrus
G$\_$front$\_$inf-Orbital	Orbital part of the inferior frontal gyrus
G$\_$Ins$\_$lg$\_$and$\_$S$\_$cent $\_$ins	Long insular gyrus and central sulcus of the insula
G $\_$insular $\_$short	Short insular gyri
G $\_$oc-temp $\_$med-Lingual	Lingual gyrus, ligual part of the medial occipito-temporal gyrus
G $\_$rectus	Straight gyrus, Gyrus rectus
G $\_$subcallosal	Subcallosal area, subcallosal gyrus
G $\_$temp $\_$sup-G $\_$T $\_$transv	Anterior transverse temporal gyrus (of Heschl)
G $\_$temp $\_$sup-Plan $\_$polar	Planum polare of the superior temporal gyrus
G $\_$temp $\_$sup-Plan $\_$tempo	Planum temporale or temporal plane of the superior temporal gyrus
G $\_$temporal $\_$inf	Middle temporal gyrus
G $\_$temporal $\_$middle	Inferior temporal gyrus
Lat $\_$Fis-ant-Horizont	Horizontal ramus of the anterior segment of the lateral sulcus
Lat $\_$Fis-ant-Vertical	Vertical ramus of the anterior segment of the lateral sulcus
Lat $\_$Fis-post	Posterior ramus of the lateral sulcus
S $\_$calcarine	Calcarine sulcus
S $\_$cingul-Marginalis	Marginal branch of the cingulate sulcus
S $\_$circular $\_$insula $\_$ant	Anterior segment of the circular sulcus of the insula
S $\_$circular $\_$insula $\_$inf	Inferior segment of the circular sulcus of the insula
S $\_$circular $\_$insula $\_$sup	Superior segment of the circular sulcus of the insula
S $\_$collat $\_$transv $\_$ant	Anterior transverse collateral sulcus
S $\_$interm $\_$prim-Jensen	Sulcus intermedius primus (of Jensen)
S $\_$oc-temp $\_$med $\_$an$\_$Lingua	Medial occipito-temporal sulcus and lingual sulcus
S $\_$orbital $\_$lateral	Lateral orbital sulcus
S $\_$orbital $\_$med-olfact	Medial orbital sulcus
S $\_$orbital-H $\_$Shaped	Orbital sulci
S $\_$pericallosal	Pericallosal sulcus
S $\_$postcentral	Postcentral sulcus
S $\_$precentral-inf-part	Inferior part of the precentral sulcus
S $\_$suborbital	Suborbital sulcus (sulcus rostrales, supraorbital sulcus)
S $\_$subparietal	Subparietal sulcus
S $\_$temporal $\_$transverse	Transverse temporal sulcus

.

## Footnotes

1 Figure produced by open source visualization software Visbrain (Combrisson et al. 2019).

2 Figure produced by Brainstorm software (Tadel et al. 2011).

## Code Availability

The code comprises our open-access code IAS-MEG, available at https://github.com/IAS-code/IAS-MEG, and custom Matlab functions for constructing BR-ALI vectors and random samples of power spectral densities and for performing LDA; these functions are available upon direct request. Conditions for sharing or re-use include citation of related papers and comply with CCBY-NC-ND 4.0 licensing.
